# The Role of Extracellular DNA and Histones in Ischaemia-Reperfusion Injury of the Myocardium

**DOI:** 10.1007/s10557-020-06946-6

**Published:** 2020-02-15

**Authors:** Mohammed Shah, Derek M. Yellon, Sean M. Davidson

**Affiliations:** The Hatter Cardiovascular Institute, 67 Chenies Mews, London, WC1E 6HX UK

**Keywords:** Myocardial infarction, DAMPs, Innate immunity, DNA, Pyroptosis

## Abstract

Despite an increase in the rates of survival in patients suffering myocardial infarction, as yet there is no therapy specifically targeting ischaemia and reperfusion injury of the myocardium. With a greater understanding of immune activation during infarction, more potential treatment targets are now being identified. The innate immune system is believed to play an important role in the myocardium after ischaemia-driven cardiomyocyte death. The release of intracellular contents including DNA into the extracellular space during necrosis and cell rupture is now believed to create a pro-inflammatory milieu which propagates the inflammatory process. DNA and DNA fragments have been shown to activate the innate immune system by acting as Danger-Associated Molecular Patterns (DAMPs), which act as ligands on toll-like receptors (TLRs). Stimulation of TLRs, in turn, can activate intracellular cell death pathways such as pyroptosis. Here, we review the role of DNA fragments during ischaemia and reperfusion, and assess their potential as a target in the quest to preserve cardiomyocyte viability following myocardial infarction.

## Targeting Cell-Free DNA in Myocardial Infarction

Despite our increasing understanding of the pathogenesis of myocardial infarction, it remains a leading cause of premature death in the Western world [1, 2]. The widespread adoption of percutaneous coronary intervention has resulted in a significant reduction in the duration of coronary ischaemia once the clinical diagnosis of coronary artery occlusion and ST elevation myocardial infarction (STEMI) has been made. Restoring blood flow to the myocardium promptly can prevent excessive cardiomyocyte death. Conversely, delaying treatment is associated with worse outcome and death. As a consequence of the success in instigating rapid reperfusion therapy, there has been an increase in the survival of STEMI patients, but this has led to a greater incidence of subsequent heart failure [3]. Paradoxically, both ischaemia and the subsequent reperfusion lead to excessive cardiomyocyte death which results in profound “remodelling” of the heart associated with fibrotic replacement of the myocardial cytoskeleton, altering the geometry of the ventricle and resulting in impaired pump function and heart failure—also called ventricular remodelling [4].

**Fig. 1:**
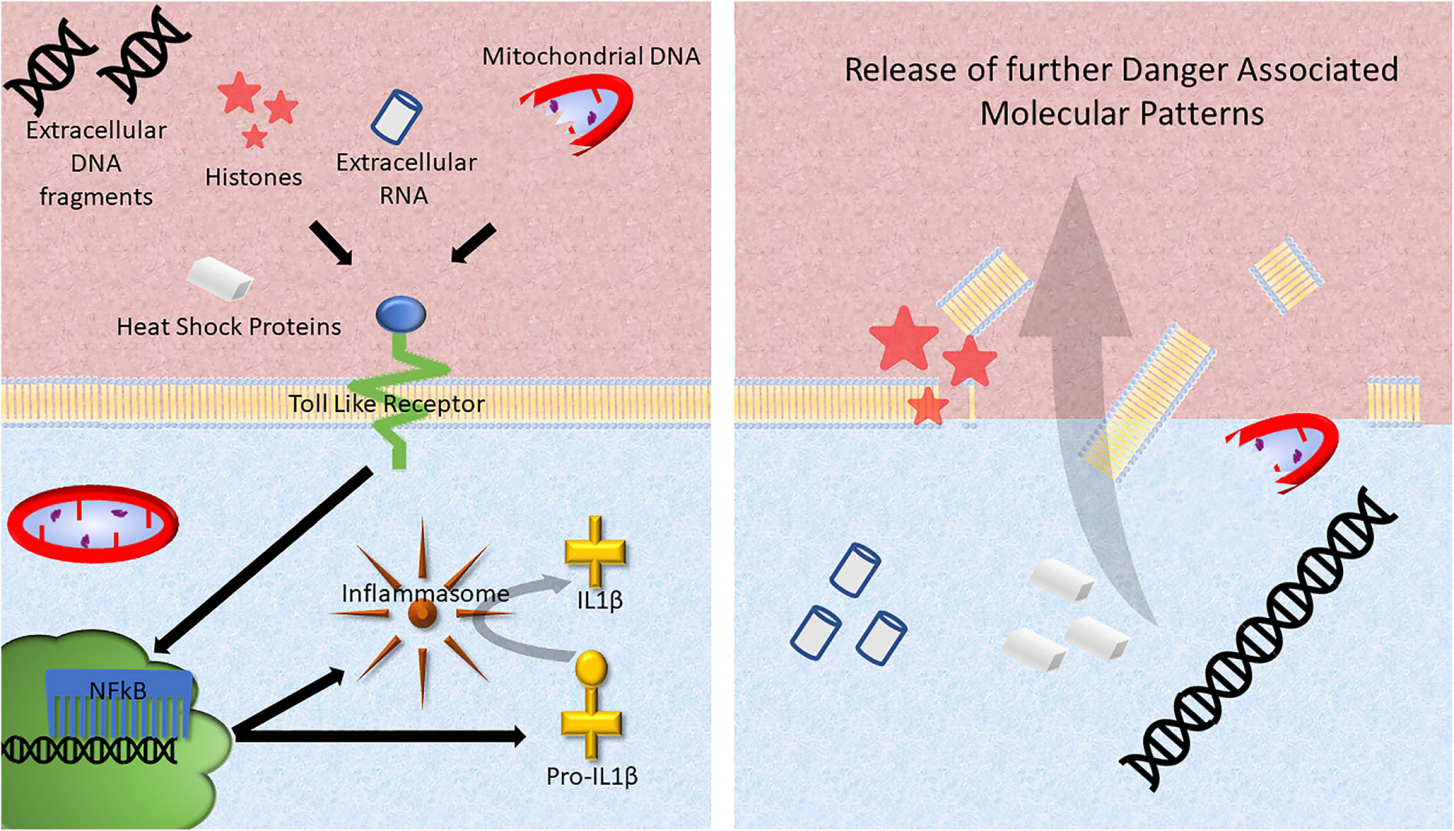
During necrosis, the cell membrane breaks down and the fragmented intracellular contents enter the extracellular space. Here, certain components such as DNA, heat shock proteins and histones can act as danger-associated molecular patterns (DAMPs), further activating intracellular cell death pathways via toll-like receptor (TLR). TLRs trigger an intracellular signaling cascade that culminates in the translocation of NF-κB to the nucleus where it stimulates the synthesis of proteins including the components of the inflammasome complex, pro-IL-1β and pro-caspase-1. Inflammasome activation is dependent on a secondary signal. Extracellular DAMPs such as ATP can trigger K^+^ efflux, triggering the formation and activation of the inflammasome complex. This facilitates autocatalytic activation of pro-caspase-1 into caspase-1 and cleavage of the pro-IL-1β into IL-1β. The active caspases contribute to pyroptosis and cell membrane rupture. The subsequent release of intracellular contents including DNA into the extracellular space results in this debris functioning as additional DAMPs, thereby propagating a wave of cellular injury and death

In order to help preserve cardiomyocyte viability after ischaemia-reperfusion injury, attention has focussed for many years on trying to understanding the process of cardiomyocyte death and inflammation after myocardial reperfusion [5–8]. There is increasing evidence that inflammation induced by ischaemia-reperfusion may actually contribute to cardiomyocyte death, excessive scar formation, and poor ventricular remodelling [9, 10]. Unfortunately, the results of the majority of clinical trials into the use of anti-inflammatory therapies for treating MI have been disappointing, illustrating our lack of understanding of ischaemia-reperfusion-induced inflammation in the myocardium. One important target is the process by which cardiomyocytes, which are viable at the point of reperfusion, die during reperfusion. This type of cell death differs from phagocytosis or apoptosis in that it is more uncontrolled and results in the rupture of the sarcolemma and release of the intracellular contents into the extracellular space. Current well-established cardioprotective strategies such as ischaemic preconditioning preserve cardiomyocytes during ischaemia-reperfusion injury, thereby limiting the release of intracellular debris [10]. During this process, the dying cells propagate the inflammatory response throughout the reperfusion zone, as the intracellular debris act as Danger-Associated Molecular Patterns (DAMPs) which are ligands for activation of the innate immune system [11] (Fig. 1). This type of cell death is called “necrosis”. Examples of DAMPs include mobility group box-1 protein (HMGB1), heat shock proteins, adenosine, extracellular RNA, mitochondrial DNA, and interleukin (IL)-1α all of which may stimulate the innate immune response. Recently, it has been shown that cells can also undergo a type of programmed necrosis, referred to as “necroptosis” or “pyroptosis” [12–14]. Identifying and targeting these DAMPs has provided varying results in attempts to save myocytes from the deleterious effects of reperfusion. This is often difficult because many identified DAMPs such as HMGB1 have complex, multifaceted roles, and inhibiting their function may instead be detrimental as inflammation is important in the process of cardiac repair after an insult. One potential DAMP that may be a promising target is DNA itself, which has the benefit of having no such multifaceted effect once outside the cell. There is now some evidence to suggest that cell death may be propagated by intracellular material such as DNA in an extracellular environment, contributing to excessive myocyte death in the myocardium after ischaemia-reperfusion injury [15–18]. One potential DAMP that has been highlighted is DNA and its components. This review aims to highlight this potentially promising target for future cardioprotective therapies.

## Inflammation and Ischaemia-Reperfusion Injury

Ischaemia causes the cardiomyocytes to switch to anaerobic glycolysis to generate ATP, but this increases lactate production which causes a rapid drop in intracellular pH, driving ion exchangers to extrude protons at the expensive of accumulating intracellular Na and subsequently Ca ions. While the acidic conditions inhibit the opening of the MPTP (mitochondrial permeability transition pore), the cytosolic calcium overload results in cardiomyocyte hypercontracture. With reperfusion, the arrival of oxygen permits the re-activation of the electron transport chain, but also results in a burst of ROS (reactive oxygen species) production. In combination with Ca overload, ROS induce the opening of the MPTP, depleting ATP and damaging intracellular structures. This can either directly cause cellular death via necrosis or can cause extensive damage to cell membranes by lipid peroxidation and enzyme denaturation as well as direct oxidative damage to DNA, such that the resultant non-viable cell activates intracellular cell death signalling pathways including pyroptosis. As a consequence, the necrotic or pyroptotic cell releases its intracellular contents into the extracellular milieu. At a later time-point, several hours after reperfusion, the pro-inflammatory milieu attracts neutrophils which invade the necrotic region [10, 14, 19, 20].

During the process of reperfusion, there is an intense inflammatory process activated within the ischaemic area, coordinated by the huge influx of immune cells that circulating blood brings with it. A crucial aspect of the activated innate immune response is leukocyte infiltration into the necrotic myocardium. Leukocytes, the vast majority of which reside in the circulation or lymphatic tissue, play an important role during the process of myocardial infarction and repair. Neutrophils are the most abundant leukocyte and they infiltrate the injured myocardium early during ischaemia [21, 22]. Within the milieu of the infarcted myocardium, they exert direct effects such as tissue infiltration [23, 24], proteolysis, generation of oxygen free radicals [25], the release of pro-inflammatory cytokines, and stimulation of the complement cascade [26]. The resulting immune cascade is beyond the scope of this review; however, its complexity and the presence of multifunctioning mediators are undoubtedly why an effective anti-inflammatory therapy is yet to be developed.

## Targeting Inflammation to Reduce MI Injury

There have been attempts to target the immune response during and after an infarction; however, the results of the majority of clinical trials have been disappointing. Earlier attempts were focussed on using corticosteroids, an idea borne out of cell-based studies showing that they conferred protection upon cardiomyocytes during ischaemia [27]. However, despite this promising theory, clinical trials gave conflicting results and in some cases raised serious concerns into the use of corticosteroid therapy post-infarction [28, 29]. Indeed, there is growing evidence that corticosteroids may impede fibroblast function and thus prevent healthy repair [30].

As understanding of the immune process has improved, more specific immunomodulatory therapies have been trialled in the setting of ACS. Crucial to neutrophils entering the ischaemic zone is the interaction of circulating neutrophils and the vascular endothelium. One way to block this step is through inhibiting the binding of leukocyte surface adhesion molecules such as P-selectin [31–33]. Inclacumab, a P-selectin inhibitor, was used in a randomised trial of 544 NSTEMI patients, but the inconclusive results only showed a trend towards a reduction in troponin levels in the treatment arm [34]. The HALT-MI study looked at antibody-mediated inhibition of the CD11/CD18 integrin receptor on leukocytes in 420 patients with an acute MI; however, treatment with the antibody to the integrin did not reduce infarct size [35]. Tocilizumab, the antibody to IL-6 receptor, reduced levels of troponin during in-hospital stay in 117 NSTEMI patients [36]. The APEX-AMI trial looked at using Pexelizumab, a humanised monoclonal antibody that binds to C5 component of the complement cascade, during PCI after myocardial infarction. A total of 5745 patients were recruited but there was no difference in all-cause mortality after 90 days between the treatment and non-treatment group [37].

Despite these disappointing findings, the search for an effective anti-inflammatory therapy for acute MI continues. The exciting results of the recent CANTOS trial in the setting of atherosclerosis have reignited the hope of developing an equivalent effective immunomodulatory therapy for ischaemia-reperfusion injury. In the CANTOS trial, Canakinumab, a human monoclonal antibody targeting interleukin 1 beta (IL-1β), was administered to patients who had previously suffered a myocardial infarct and raised circulating CRP levels. Treatment demonstrated a 15% reduction in relative risk for the composite primary endpoint of non-fatal MI, non-fatal stroke, and death from cardiovascular disease [38].

A huge body of animal studies offer further tantalising hope of identifying new treatment targets in preventing the inflammatory damage in ischaemia-reperfusion injuries. Modulating pro-inflammatory cytokines in animal models have yielded exciting results; IL-1β blockade in a mouse ischaemic cardiomyopathy model significantly improved LV function [39]. Similar studies targeting IL-2 or IL-18 in animal models have demonstrated significant reduction in cardiomyocyte death and improvement in LV function [40, 41]. Chemokines [42], inflammasomes, and TGF-β [43] are some of the other targets that have demonstrated potential benefit in animal models of ischaemia-reperfusion injury.

However, one avenue that has received limited coverage as a potential new target is the role of extracellular DNA and the innate immune system. Innate immunity is the first line of defence to tissue injury and represents an evolutionary older branch in comparison with the more specific and targeted adaptive immunity. The innate immune system first appeared 750 million years ago and has been remarkably conserved throughout the evolutionary tree of life [44, 45]. A key initiator of an innate immune response is immune stimulation via a DAMP [11]. Therefore, identification of DAMPs released during ischaemia-reperfusion injury may lead to the identification of a valuable drug target. The appearance of excessive amounts of intact, high-molecular-weight, extracellular DNA is one of many differences between controlled cell death pathways such as apoptosis and uncontrolled necrotic cell death [46, 47]. When cells undergo apoptosis, intracellular DNA is methodically degraded and shielded from the immune system by retention within plasma membrane vesicles (apoptotic bodies). However, during ischaemia, cells die primarily via a process of necrosis during which DNA is released into the extracellular space and blood. The nucleus of every eukaryotic cell (except red blood cells, which do not have a nucleus) contains approximately 6 pg of DNA. Therefore, since the human myocardium contains approximately 5 billion cells, a large left ventricular myocardial infarct could cause the death of ~ 1 billion cardiomyocytes which can potentially release ~ 1 mg of DNA and DNA fragments into the extracellular space [48, 49]. This huge quantity of DNA is then free to diffuse within the necrotic milieu of the infarct zone.

Another large source of extracellular DNA during ischaemia and reperfusion comes from infiltrating leukocytes. In response to a TLR-dependent process, neutrophils, the most abundant leukocytes in the myocardium during reperfusion, discharge their nuclear DNA forming an extracellular net of DNA rich in histones [50]. This process, termed NETosis, ultimately kills the neutrophil whilst it lays down a histone-rich mechanical mesh which traps debris. NETs (neutrophil extracellular traps) can break down and release histones causing further damage to tissue remote from the initial necrotic site [51]. The NETs also play a crucial role in thrombosis, platelet aggregation, and occluding blood vessel further exaggerating coronary ischaemia [52]. Thrombi aspirated from the coronary arteries of patients who suffered STEMI demonstrate that the burden of NETosis positively correlates with infarct size and negatively correlates with ST segment resolution [53].

The crucial role of DNA breakdown in normal physiology is highlighted by the fact that mice lacking the DNA cleaving enzyme DNAse II die shortly after birth [54]. Furthermore, deficiencies in the normal biological process of DNA digestion and processing are linked to diseases with inappropriately active innate immune systems or autoimmunity [55–57]. Elevated levels of circulating DNA are also associated with a variety of conditions from trauma, tumour malignancy, and sepsis, all of which are themselves associated with a degree of immune activation or inflammation [58, 59]. Each DNA nucleosome core consists of superhelical DNA wound around an octamer of histones, composed of two copies of each of the core histones H2A, H2B, H3, and H4 [60]. The linker histone H1 binds to the complete nucleosome core particle and forms higher order structures [61]. After a necrotic event, cellular DNA may be released either as DNA fragments or as nucleosomes, both of which are well-recognised DAMPs [18]. The extent to which mammalian DNA components are cytotoxic was first investigated by Xu et al., who showed that intravenous injection of isolated histones into mice caused death through sepsis within minutes [62]. Curiously, administration of intact nucleosomes did not have this effect. It has now been shown that this effect is mediated through the toll-like receptors TLR2 and TLR4, two crucial receptors involved in activation of the innate immune system [63]. Unlike human DNA, foreign microbial DNA has also long been accepted as a potent stimulator of the innate immune system [64]. A heterogeneous group of pattern recognition receptors on immune cell surfaces detects foreign microbial nucleic acids, including TLR3, TLR7–TLR9 [65, 66].

A certain quantity of cell-free, circulating DNA is understood to be part of the normal physiological state in both humans and rodents; its concentration is tightly controlled by extracellular DNAases [67]. It is now believed that during normal cellular process such as cell division, the amount of extracellular DNA remains manageable through continuous degradation to maintain a level below the immuno-stimulatory threshold [68, 69]. If, however, the threshold is breached in conditions such as necrotic cell death, the DNA may act on the same pathogenic receptor pathways stimulating an innate immune response [70–72]. A well-documented finding in auto-inflammatory conditions is the presence of circulating cell-free DNA incorporated into immune complexes [73], confirming the ability of DNA to act as an auto-antigen. Previously, it was believed that the presence of unmethylated CpG dinucleotides in microbial DNA conferred foreign DNAs ability to interact with TLR9 and stimulate the innate immune response. Mammalian purified DNA dinucleotides are mostly methylated so in theory they should not exhibit an immuno-stimulatory response, but mammalian complexed DNA, either within histones or DNA-binding proteins, has been demonstrated to induce TLR9-mediated signalling [74, 75]. Extracellular mammalian DNA has been shown to have this effect both by interacting with TLR9 [76] to activate the innate immune system and by TLR-independent mechanism which increase the transcription of type 1 interferons a potent pro-inflammatory cytokine [77–79]. Unlike naked DNA, cell-free chromatin contains abundant proteins that may expose epitopes for helper T cells to identify them as foreign. Indeed, the appearance of antibodies to chromatin precedes the occurrence of anti-DNA antibodies suggesting chromatin plays a crucial role in developing an auto-inflammatory response to self-DNA [59].

## The Role of Extracellular Histones in Innate Immunity

Apoptotic or necrotic cells induced by ischaemia release histones [80] either as part of nucleosome fragments or on their own. These extracellular histones have also been shown to trigger inflammation and cell death, either by stimulating pro-inflammatory cytokines resulting in the activation of cell death pathways or through the process of neutrophil extracellular traps. In human observational studies, raised histone serum levels have been demonstrated in multiple trauma patients and correlate with severity of coagulopathy, endothelial damage, and inflammation [81]. A large body of evidence demonstrates that histone-induced cell toxicity plays a crucial role in cell death during ischaemia-reperfusion injury of the myocardium. Histones are known to activate TLR2 and TLR4; furthermore, TLR knockout mice are protected from the lethal effects of histones [63]. In an ischaemic stroke model, histone infusion is correlated with large infarct size and conversely histone neutralisation via an antibody infusion results in a reduction in infarct size [82]. In a toxic liver injury model, free histones mediated cytotoxicity of liver cells via a TLR-dependent process—an effect that was abrogated by anti-histone antibodies [63]. Furthermore, in liver cells, histone-stimulated TLR activation results in activation of the intracellular NLRP3 inflammasome and subsequent pyroptosis [83]. Histones have been shown to mediate endothelial cell cytotoxicity, resulting in acute lung haemorrhage, thrombosis, and oedema [84]. Similar cytotoxic effects of histones have been demonstrated in kidney injury [85], sepsis [62], and even hair follicle death [86]. There is also increasing evidence that histones can cause cytotoxicity independent of immunostimulation, damaging endothelial cells and stimulating an influx of intracellular calcium and subsequent necrosis [84]. Thus, increasing evidence suggests free histones function as DAMPs, leading to both inflammatory and toxic responses culminating in cell death.

## DNA and Cell Death (Inflammasome Activation and Pyroptosis)

It has been shown that extracellular DNA and histones could function as an alarmin or DAMP by causing activation of TLRs. As well as playing a crucial role in the innate immune system, activation of TLR can activate cell death pathways such as pyroptosis. It is now believed that during ischaemia and reperfusion, pyroptosis may contribute to infarct size and subsequent poor remodelling of the myocardium [12, 87, 88]. This has culminated in a great deal of interest in targeting intracellular cell death pathways to limit the degree of cell death during myocardial infarction [8, 89, 90].

During ischaemia, necrotic cell debris including DNA fragments circulate in the extracellular matrix creating a pro-inflammatory milieu. Activation of the TLRs on surviving cardiac cells in the border zone of the infarct area leads to activation of downstream intracellular signalling pathways, which convene to result in NF-κB mediated expression of the protein components that make up the NLRP3 inflammasome [91, 92]. Following a secondary trigger, the individual protein components which now exist in the cytoplasm of the cell aggregate to form a multiprotein oligomer also called the inflammasome complex [93, 94]. This complex is now able to interact with pro-caspase-1 and leads to its conversion into its active caspase-1 form [95]. Caspase-1 begins the subsequent autocatalytic activation of the pro-inflammatory cytokines IL-1β and IL-18 [96]. An additional substrate of caspase-1 is the cytosolic protein, gasdermin D (GSDMD) [97–99]. Following cleavage by caspase-1, the N-terminal fragments of GSDMD (GSDMD-N) oligomerise within the cell membrane to form pores. These pores result in loss of cell membrane integrity, leading pyroptotic cell death [100]. The pores also increase membrane permeability to IL-1β and IL-18 leading to their extracellular release, and these cytokines amplify the inflammatory response and mediate further injury [42, 87, 88, 101–108].

## Evidence for a Role of Extracellular DNA in Ischaemia-Reperfusion Injury of the Myocardium

A number of animal studies have proven that self-DNA may be an effective target to inhibit inflammation and myocyte death during ischaemia-reperfusion injury. In a murine model, it was demonstrated that histones caused cardiomyocyte toxicity and an in vivo heart ischaemia-reperfusion model, DNAse 1 treatment, disrupted extracellular cytotoxic chromatin resulting in a reduction myocardial histone concentration [17]. This correlated with a significant improvement in left ventricular remodelling and cardiomyocyte survival. Ge et al. supported this finding in a murine model of ischaemia-reperfusion showing that DNAse with the addition of recombinant tissue-type plasminogen activator resulted in a reduction of infarct size as well as decreasing the density of neutrophil-associated NETs [109]. This also leads to an improvement in left ventricular remodelling. Curiously, this effect was not observed when DNAse or rt-PA was administered on its own [109]. Savchenko et al. administered DNAse to PAD4^-/-^ mice which do not produce NETs as well as wild type mice [110].The study demonstrated that myocardial ischaemia-reperfusion injury caused an increase in nucleosomes, neutrophil infiltration, and histone H3 at the site of injury. Treatment with DNAse improved cardiac contractile function to a similar degree in both wild type and PAD4^-/-^ deficient mice. This suggests that DNA fragments contribute to cardiomyocyte dysfunction during reperfusion irrespective of NETs, possibly by acting as a DAMP. Using an in vivo rat model, Downey’s group have made similar findings, demonstrating that DNAse administered after 30 min of coronary artery occlusion resulted in a significant reduction in infarct size [111]. Interestingly, the addition of mitochondrial DNA inhibitor with DNAse resulted in a greater reduction in infarct size then that which was seen with DNAse alone [111]. This would suggest that nuclear DNA could be acting through a different pathway than the well-recognised DAMP, mitochondrial DNA.

Endothelial dysfunction plays a crucial role in ischaemia-reperfusion injury, contributing to myocardial stunning, microvascular obstruction, and exposing the myocytes to toxic stimuli which contribute to lethal myocardial injury [112–114]. Heparin consists of a high volume of negatively charged sulphated proteoglycans, binds to histones, and inactivates them through high-affinity electrostatic interactions [115]. It has long been shown that heparin protects the coronary endothelium and myocardium from ischaemia-reperfusion injury [116–119]. Both heparin and chondroitin sulphate have both been shown to protect vascular endothelial cells from histone-induced cytotoxicity in vitro [120, 121]. Furthermore, heparin derivatives reduce infarct size in a rat model of ischaemia-reperfusion by inhibiting caspase-dependent, cell death pathways [122].

## Summary

In the quest to protect cardiomyocytes from the deleterious effects of ischaemia-reperfusion injury, identification of pyroptosis as a contributing factor to infarct size has revealed a target for future cardioprotective therapies. Extracellular DNA fragments from dead cells and neutrophils are potent instigators of TLR-dependent inflammasome activation and subsequent pyroptosis. The use of DNAse and DNA-neutralising therapies has shown some promise in animal models of preventing the damaging effects of ischaemia-reperfusion injury. Identification and targeting the instigators of pyroptosis may provide benefit in limiting cell death post-infarction and preventing the morbidity and mortality associated with ischaemia and reperfusion injury.
